# Adverse effects of xenogenic scaffolding in the context of a randomized double-blind placebo-controlled study for repairing full-thickness rotator cuff tears

**DOI:** 10.1186/s13063-019-3504-3

**Published:** 2019-07-01

**Authors:** José Ramón Lamas, Carlos García-Fernández, Pilar Tornero-Esteban, Yaiza Lópiz, Luis Rodriguez-Rodriguez, Luis Ortega, Benjamín Fernández-Gutiérrez, Fernando Marco

**Affiliations:** 1 UGC de Reumatología, Hospital Clínico San Carlos, Instituto de Investigación Sanitaria del Hospital Clínico San Carlos (IdISSC), 28040 Madrid, Spain; 2 UGC de Cirugía Ortopédica y Traumatología, Hospital Clínico San Carlos, Instituto de Investigación Sanitaria del Hospital Clínico San Carlos (IdISSC), Madrid, Spain; 3 Servicio de Anatomía Patológica Hospital Clínico San Carlos (IdISSC), Madrid, Spain

**Keywords:** Xenogenic matrix, Mesenchymal stem cells, Clinical trial, Comparative effectiveness, Functional status, Rotator cuff

## Abstract

**Purpose:**

The purpose of the study was to compare the safety and efficacy of autologous mesenchymal stem cells (MSCs) embedded in a xenogenic scaffold for repairing the supraspinatus tendon.

**Methods:**

This was a randomized, double-blind and placebo-controlled trial evaluating patients with full-thickness rotator cuff tears (Eudra-CT, 2007–007630-19). Effectiveness was evaluated using the Constant score and a visual analogue pain scale (VAS).

Constant score has four domains including pain (15 possible points), activities of daily living (20 possible points), mobility (40 possible points), and strength (25 possible points). Scores range from 0 points (most disability) to 100 points (least disability).

The structural integrity of the repaired tendon was assessed by magnetic resonance imaging (MRI) according to Patte and Thomazeau classification criteria.

The primary study end point was an improvement in the Constant score by 20 points at one year compared to initial assessment.

**Results:**

The trial was stopped due to adverse effects observed in both groups. Only thirteen patients were included and analyzed. The Constant questionnaire showed a significant improvement in the MSC treatment group compared with the preoperative data (p = 0.0073). Secondary outcome measures were similar in both groups.

**Conclusions:**

Our study showed preliminary inconclusive clinical outcomes in the patients treated with MSCs. Adverse events revealed the need for further approaches using scaffolds of a different nature or perhaps no scaffolds, in the context of small joints.

**Trial registration:**

Eudra-CT, 2007-007630-19. Registered on 30 January 2008.

**Level of evidence:**

A Level 1 of evidence treatment study.

**Electronic supplementary material:**

The online version of this article (10.1186/s13063-019-3504-3) contains supplementary material, which is available to authorized users.

## Background

The shoulder is the most mobile joint in the body, providing multiple degrees of motion. Shoulder biomechanics are based on the interaction of multiple structures perfectly adjusted to provide its function. Particularly, the rotator cuff, an anatomical term defining a set of muscles and tendons, is responsible for shoulder stabilization, centring of the humeral head in place and mobility and participating in the abduction and external rotation movements that lift and rotate the humerus [36]. Defects or lesions in this structure can cause considerable tissue damage including cuff tendon ruptures, pain associated with shoulder motion, edema, inflammation and disability.

Rotator cuff tear is a common shoulder disease increasing with age and previous trauma. Its prevalence ranges between 4% in asymptomatic patients below 40 years and 54% in patients over 60 years of age [9]. Tears are the consequence of trauma or they develop gradually due to mechanical and/or inflammatory processes. They cause pain, shoulder weakness and upper extremity disability. Medical management of these symptoms includes the use of conservative treatments with nonsteroidal anti-inflammatory drugs (NSAIDs), corticosteroid injections and physiotherapy [21].

Despite the improvement in surgical techniques, tendon function is often unrecoverable. Currently there is no clear evidence supporting or refuting the efficacy of current surgical interventions for rotator cuff tears [15]. In fact, outcomes vary tremendously, with failure rates as high as 25%, 35% and even 90% depending on the tear size and follow-up time [1, 7, 17, 22, 23, 38]. These disappointing outcomes highlight the need for alternative therapeutic approaches allowing better restoration of tendon functionality after injury.

Tendons are capable of self-regeneration but this capability is limited when the defects to be repaired are extensive. In addition, repair challenges are highly influenced by mechanical loads on this anatomical structure, and often, by degeneration in a tendon at the time of surgery. The goal of treatment, particularly in the context of regenerative medicine, has focused on augmenting suture fixation with several biologic collagen-rich scaffolds such as human dermal allografts [2], crosslinked acellular porcine patches [12], and other bioengineered commercial extracellular matrix materials [14]. These grafts increase the suture strength and provide a similar biochemical composition to that of the tendon; however, they fail to improve rotator cuff tendon healing or its biomechanical functions. This failure has been attributed to differences in elasticity between grafts and native tendons.

Other strategies carried out using growth factors, plasma rich in growth factors or fibrin clots promote tissue regeneration but they do not provide clear biomechanical benefits when compared to untreated controls [29].

Autologous tissue-specific cells are the gold standard for cell therapies to overcome the limited capacity for self-regeneration of tendons, particularly in rotator cuff ruptures; however, isolation of tenocytes in adequate numbers is difficult, due to their highly dense tendon extracellular matrix (ECM). Other cell sources are necessary for tendon repair and mesenchymal stem cells (MSCs) have been proposed as the best source [26, 28, 30]. MSCs have multipotent differentiation in cells of mesenchymal lineage and with their observed reparative effects in many clinical and preclinical models, suggesting they are crucial in injury healing as well as modulating the immune response [11, 32]. Clinical application of MSCs in animal models of tendon healing has also been subject of research by our group and others, reporting the benefits of MSCs in relation to the biomechanical and histological properties of tendons [5, 31, 34].

However, despite the apparent advantages of MSCs in animal models of tendon repair, there are only preliminary results reported in humans [19]. Therefore, the aim of this study was to assess the ability of MSCs to repair rotator cuff tendon injuries in humans, to enhance shoulder function and the patient’s perception of improvement. For this purpose, we conducted a controlled and randomized trial combining, or not, autologous MSCs augmented with a commercially available xenogeneic ECM of type I collagen.

## Methods

### Study design and patients

This was a one-year prospective, randomized, double-blind and placebo-controlled trial of patients from a single center with confirmed full-thickness rotator cuff tears. We included 32 eligible patients (48–66 years old) in the initial design of the study. Patients were enrolled consecutively (from January 2011 to November 2012) after simple randomization of subjects to each treatment group. Random assignment was performed using Microsoft Excel randomization functions. Inclusion criteria were established by the orthopedic surgeons involved in the study. These criteria were patients with unilateral shoulder pain and positive magnetic resonance imaging (MRI) diagnosis of full-thickness rotator cuff tear, and patients had to have been refractory to conventional medical treatment and/or rehabilitation for at least 3 months. All patients enrolled gave their written informed consent and the study was approved following the guidelines of the institutional ethics committee (Comité Ético de Investigación Clínica Hospital Clínico San Carlos–Madrid).

The exclusion criteria were rheumatic disease, glenohumeral osteoarthritis, fractures, diabetes mellitus, infections or tumors. The treatment group was composed of patients treated with 20.10^6^ autologous bone marrow (BM)-MSCs in combination with a type I collagen membrane (OrthADAPT™). The control group was composed of patients treated only with type I collagen membrane.

### Outcome measures

Functional results were assessed by the Constant score and classified according to the Walch and Marechal Constant Index classification [10]. The structural integrity of the repaired tendon was evaluated by MRI and classified according to the Patte and Thomazeau criteria. Patients’ perceptions of pain were measured using a visual analogue scale (VAS), ranging from 0 to 10. The study was registered at the European Union Drug Regulating Authorities (Eudra-CT, 2007-007630-19) and was in accordance with ethical standards for research on human subjects.

### Bone marrow collection and MSC isolation

Bone marrow was aseptically drawn under local anesthesia from the right posterior superior iliac spine and immediately anticoagulated by heparin. The collected BM (50 ml) was processed according to Good Manufacturing Practice (GMP) guidelines in a facility at the Hospital Gregorio Marañón (Madrid, Spain). The BM-MSCs isolated from BM aspirates were expanded during a 2-week period, to obtain 20.10^6^ cells. On the day of the surgical procedure the autologous BM-MSCs were suspended in 1 ml of normal saline solution and transported to the operating room. For individuals included in the control group, the procedure was similar, but in this case the cell suspension was replaced by saline, thus ensuring that surgeons were blinded to the study allocation.

### Surgical technique

Every patient underwent a surgical procedure following conventional, open, rotator cuff repair. Briefly, following interscalene block and induction of general anesthesia, surgery was performed in the beach-chair position. An anterosuperior approach was used to reach the torn cuff.

Initial surgical procedures included acromioplasty, acromioclavicular ligament resection, bursectomy and cuff debridement. Cuff edges were mobilized and the greater tuberosity footprint was repeatedly polished until bleeding. The tendon-to-bone attachment was achieved using suture anchors (Healix, Mitek®) charged with Orthocord suture (Mitek®) and the same suture was used for any side-to-side repair needed. Once the primary repair was finished, augmentation was performed using an OrthADAPT™ bioimplant (Synovis Orthopedic & Woundcare, Inc. Irvine, CA, USA). During surgery, a 4 × 5 cm OrthADAPT™ Bioimplant was incubated for 10 min with 1 ml of saline (control) or 1 ml of suspended MSCs (treatment group) to allow cell attachment to the bioimplant. This procedure was performed without the surgeon’s knowledge of the group to which the patient belonged. The implant was then cut to the required size for the lesion augmentation. The bioimplant (either with or without BM-MSCs) was always sutured in the same fashion, facing the tendon over the repaired cuff. It was initially fixed to bone with the same sutures from the anchors used in the native cuff and then it was tensioned circumferentially around the surgical repair with 3/0 non-absorbable sutures. Wound closure was performed in the usual way by anterior deltoid fascia re-attachment to the acromion. Drains were not used. The shoulder was immobilized postoperatively with an abduction sling.

### Functional evaluation

The Constant score was applied for the injured and contralateral shoulder. Scoring items were allocated into two blocks, a subjective one, including three items (pain with a maximum score of 15, level of functional activity and hand positioning with maximum 10 points each) and an objective one, which scores different items for range of motion from 0 to 10 and muscle strength with a maximum of 25 points measured in kilograms with a portable dynamometer (Basic Force Gauge; BFG, Mecmesin). Strength testing was performed in static lateral elevation of 90° for 5 s (average of 3 attempts × 2). The results were classified according to Walch and Marechal classification criteria [37], as excellent (Constant Index 80 points or more), good (CI between 65 and 79), average (CI between 50 and 64) and poor (CI below 50). The primary study end point was an improvement in the Constant score by 20 points at one year compared to initial assessment. The 20 points of improvement were considered significant taking into account that activities of daily living as a whole had a maximum score of 20 points.

### Magnetic resonance imaging assessments

MRI evaluations were obtained preoperatively and post-operatively at 12 months. Images were reviewed independently by two experienced radiologists. These experts completed full classification of supraspinatus muscle atrophy according to the Thomazeau classification [33] and of tendon retraction according to Patte’s score [27]. Thus, patients were assigned to 3 different atrophy stages depending on the occupation ratio R defined as the ratio S1/S2 where S1 is the surface of the supraspinatus muscle and S2 is the surface of the entire supraspinatus fossa, measured on the scapular cut at the level of the medial border of the spine of the scapula. Thomazeau stages were established as stage 1, normal/slight atrophy, *R* = 1.00–0.60; stage 2, moderate atrophy, *R* = 0.60–0.40 and stage 3, severe atrophy, *R* < 0.40.

Patte’s score defines 3e stages of cuff tear retraction in the frontal plane as follows: stage 1: proximal stump close to the enthesis, or bony insertion; stage 2: proximal stump at the level of the humeral head; stage 3: proximal stump at the glenoid level.

### Statistical analyses

A required sample size of 32 subjects, 16 in each arm, was estimated, to detect differences between the two groups of at least 20 points in the means for the Constant score, with 80% power and 5% level of significance. Statistical analyses of preoperative and postoperative clinical outcome measures were evaluated by use of a non-parametric test (Mann-Whitney) using the GraphPad Prism 6 software (GraphPad Software, Inc. La Jolla, USA).

## Results

**Fig. 1:**
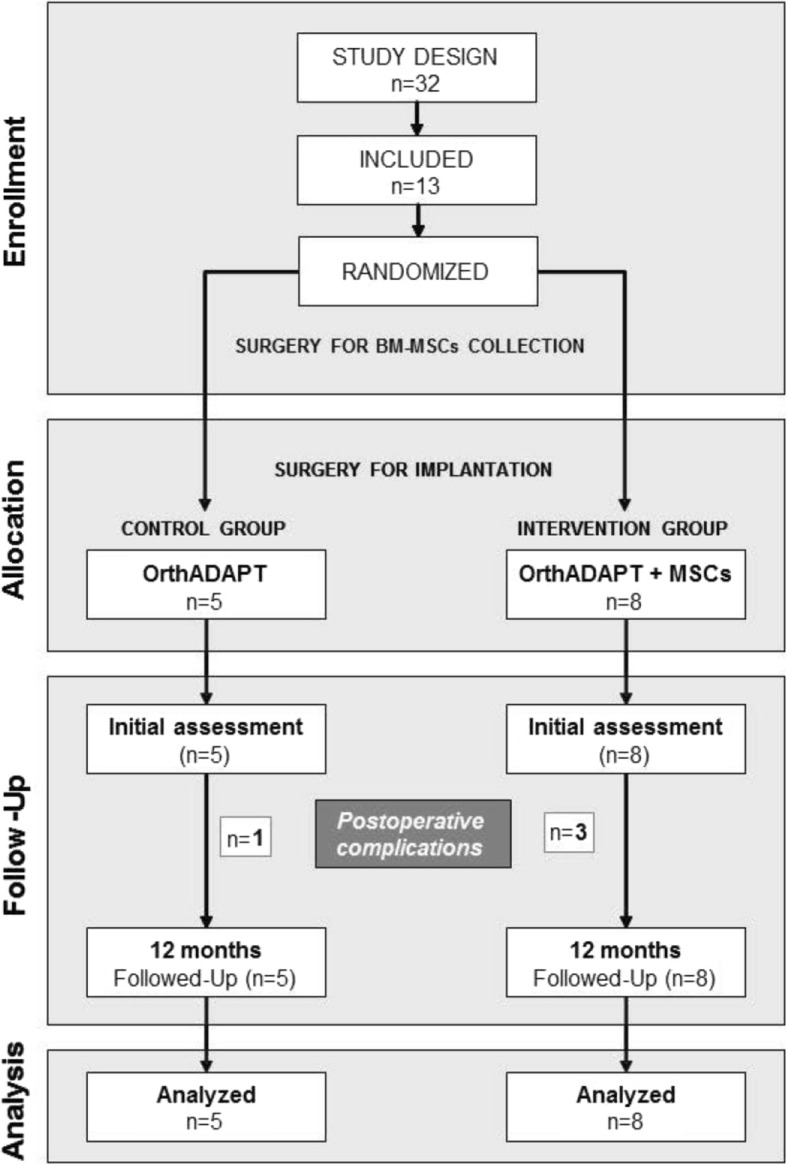
Flow chart of the patients who participated in the clinical trial. Pain and functional scores were evaluated and magnetic resonance imaging (MRI) was performed preoperatively and after one year follow up. BM-MSCs, bone marrow mesenchymal stem cells

Postoperative complications were observed in four patients and, taking into account ethical issues, we decided to finish the study prematurely (Fig. 1). Thus, of the expected 32 patients only 13 patients completed the study. The baseline characteristics of the patients are shown in Table 1. Although only 13 patients were included, there was a statistically significant improvement in the Constant score in the treatment group, (p = 0.0073) (Table 2).

**Table 1:**
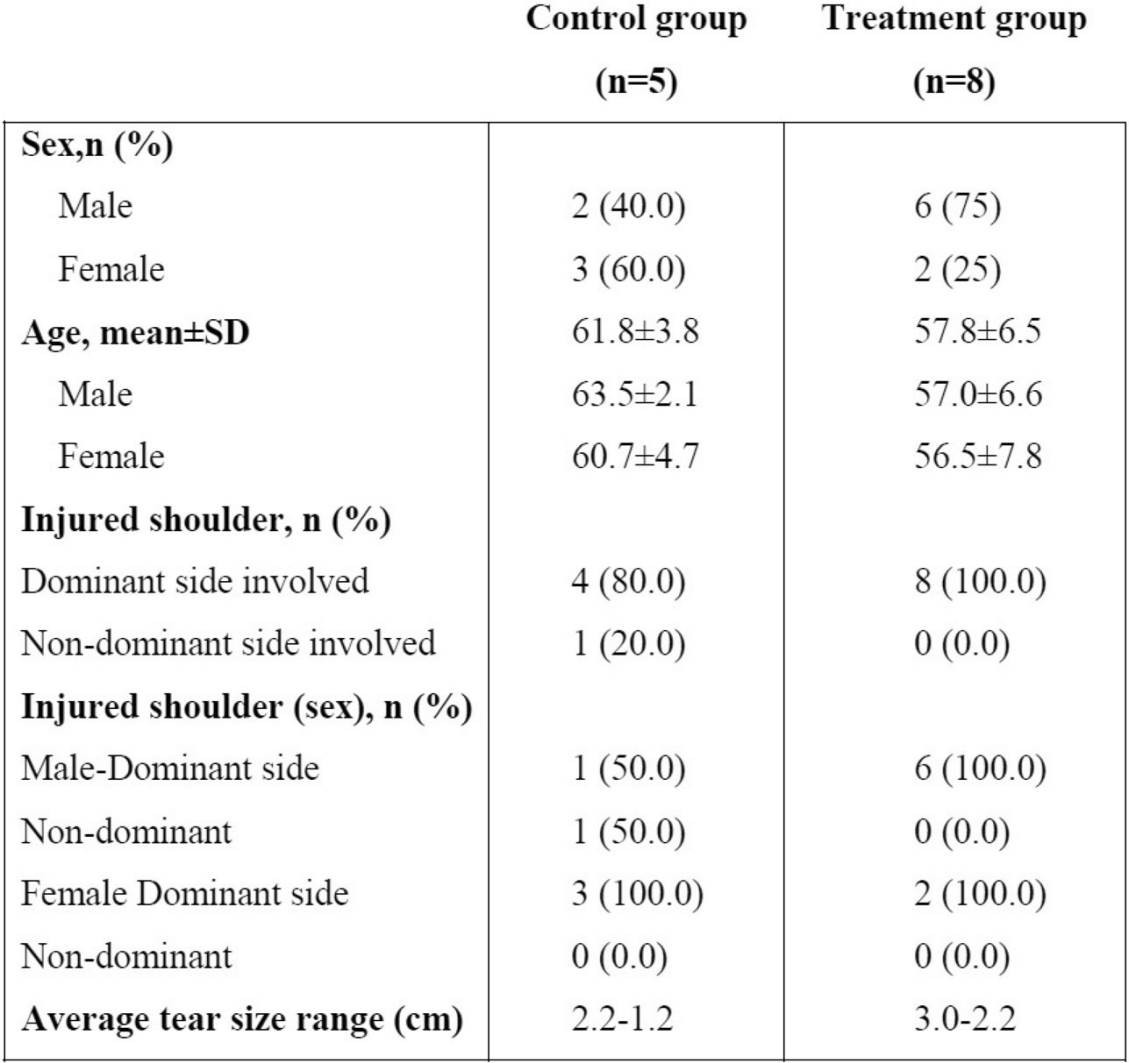
Baseline characteristics of the patients included in the analysis

**Table 2:** Visual representation of all outcomes included in the study

	Sex	Age	Shoulder involved	Preoperative					Postoperative							
				Constant		Patte’s score	Thomazeau’s score	VAS	Constant*		Patte’s Score	Thomazeau’s Score	VAS	Recurrent tear	Repair Integrity	AEs
				Contralateral shoulder	Injured shoulder				Contralateral shoulder	Injured shoulder						
Control group																
HCSC-1	Male	65	LS	93	60	1	1	8	91	33.5	1	1	1	Yes	No	No
HCSC-2	Mujer	66	RS	96	56	1	1	8	92	71	2	2	2	Yes	No	Yes
HCSC-13	Male	62	RS	75	48	1	1	9	70	66	2	2	4	Yes	No	No
HCSC-14	Female	57	RS	81	55	1	1	8	84	80	1	1	2	No	Yes	No
HCSC-15	Female	59	RS	80	37	2	2	8	80	77	1	1	2	No	Yes	No
Treatment group																
HCSC-3	Male	61	RS	65	30	3	3	9	87	63	3	3	6	Yes	No	No
HCSC-4	Male	62	RS	79	36	2	2	9	79	43	3	3	8	Yes	No	Yes
HCSC-5	Male	48	RS	94	35	2	2	8	96	62	3	3	3	Yes	No	Yes
HCSC-6	Male	60	RS	91	53	2	1	8	91	86	2	2	1	Yes	No	No
HCSC-7	Male	49	RS	89	63	1	1	7	98	87	2	2	1	No	Yes	No
HCSC-10	Female	60	RS	87	48	1	1	8	93	74	2	2	2	Yes	No	Yes
HCSC-11	Male	49	RS	89	48	1	1	8	90	79	1	1	1	No	Yes	No
HCSC-12	Female	65	RS	80	43	1	1	8	80	82	1	1	1	No	Yes	No

*AEs* adverse events, *VAS* visual analogue scale

* Statistically significant recovery between injured and contralateral shoulder in the treatment group. *P* = 0.0073

Re-rupture occurred in 3/5 patients (60%) in the control group and in 5/8 (62.5%) in the treatment group. Therefore, the rotator cuff healed in 2/5 shoulder joints (40%) in the control group compared with 3/8 shoulder joints (38%) in the MSC group.

Although the functional score was higher in the MSC-treated group, indicating a possible therapeutic advantage in the use of MSCs, the recurrence of tendon rupture and the occurrence of adverse events in 4/13 patients enrolled, forced us to prematurely terminate the study and to evaluate the causes of these undesirable complications.

**Fig. 2:**
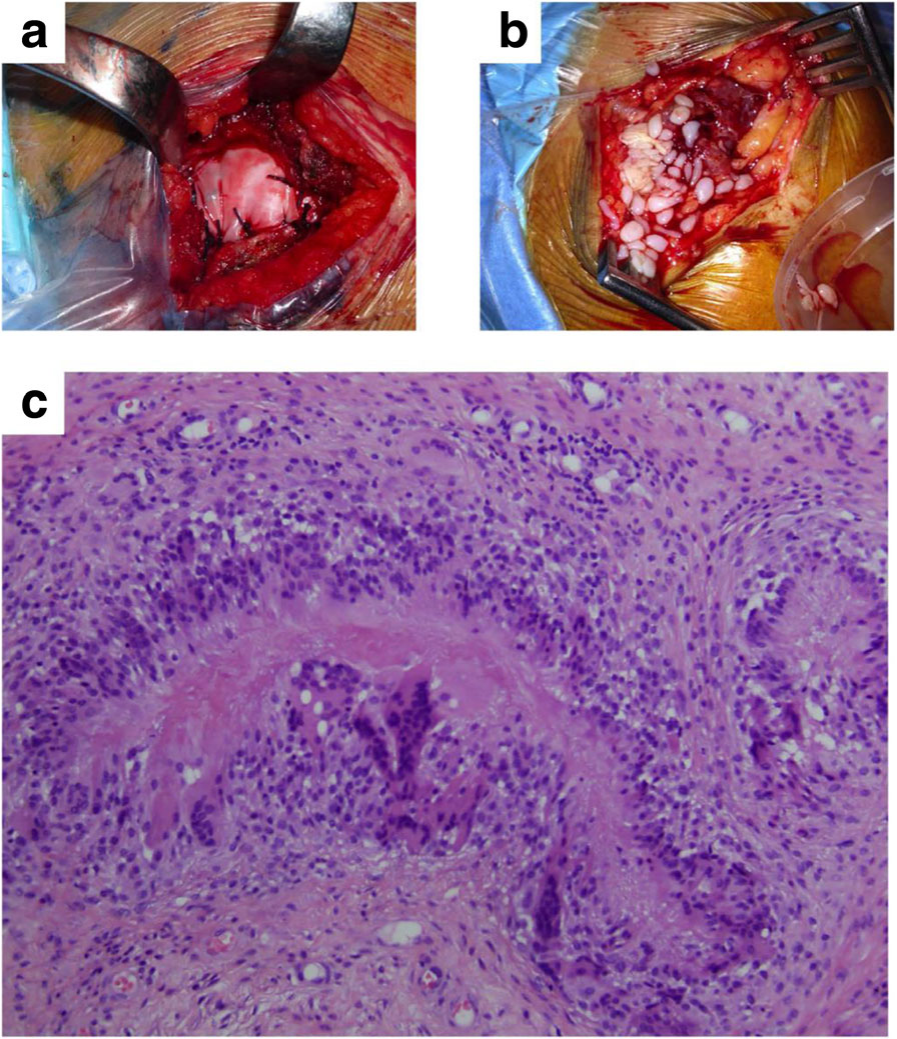
**a** Surgical scenario showing the implanted OrthADAPT™ during the initial surgery and **b** after the revision surgery to remove the inflammatory tissue. **c** Histopathological examination revealed that chronic inflammation was caused by granulomatous inflammatory changes due to a foreign-body reaction of iatrogenic etiology. The center of the image shows the foreign material engulfed and surrounded by immune cells organized into palisade

Three patients in the treatment group (23%) had postoperative complications compared to one patient in the control group (8%). The type of lesions present included the formation of supraclavicular cysts and development of subacromial inflammatory tissue (Fig. 2a and b). The exploration and the external assessment was confirmed by MRI, revealing the tendon re-rupture and fluid accumulation in the subdeltoid bursa area. Immunopathological response consisted of chronic synovitis and granulomatous lesions. In all cases, severe inflammatory response was characterized by a foreign-body-like reaction, including necrobiotic granulomas (Fig. 2c). Microbiological tests were negative in all cases.

The four patients with adverse events had tendon re-rupture. Three patients also had supraclavicular ganglions. These events were detected in three patients at the last visit (one year) and in one patient between the second (3 months) and the last visit. We decided to stop the study at the time that the third adverse event was detected. All adverse events were resolved satisfactorily after surgery, with open surgery in three patients and arthroscopic surgery in one patient. No specific clinical or demographic characteristics were observed in these patients compared with the other nine patients included (Table 2).

## Discussion

There are few reports on the therapeutic use of MSCs in shoulder surgery, both in animal and human studies [3]. Although the implantation of autologous MSCs has yielded modestly satisfactory results in the repair of musculoskeletal structures such as cartilage, bone, muscle and tendon in different animal models [5, 16, 31], the application of MSCs alone does not seem to be sufficient to improve the healing of repaired tendons, necessitating the use of scaffolds for augmentation, that is to provide resistance and maintain strength against anatomic forces in a particular histologic environment while the repair process matures. The application of MSCs for tendon repair in humans is still in the experimental phase. Several issues, such as the number and mode of MSC delivery into the surgical site and the appropriate combination of MSCs, growth factors and cytokines able to modulate the healing and/or tendon regeneration are, however, promising [6]. Moreover, the studies carried out on animal models using the OrthADAPT™ Bioimplant combined with MSCs also support the safety and efficacy of this strategy, encouraging their application in humans [34].

In recent work including 14 patients with complete rotator cuff tears, an improvement of about 19 points in the UCLA score was reported one year postoperatively when directly applying bone marrow mononuclear cells (BMMCs) in combination with a conventional repair technique [16]. Furthermore, in another study including 46 patients with patellar tendinopathy, the injection of autologous skin-derived tendon-like cells led to an improvement of around 31 points in the VISA questionnaire score after 6 months of treatment [8]. So far, there have been no clinical studies on rotator cuff repair using autologous MSCs isolated from bone marrow aspirates. Therefore, our work can be considered the first to report this approach.

In the present study, the established functional outcome was only reached in the group of patients treated with MSCs; however, there were no differences between the control and the MSC-treated group in the structural results, either in the re-tear rate or in the integrity of the repair after MRI examination. Similarly, in both groups, initial muscle atrophy and tendon retraction was maintained or continued to worsen.

Taking together, these findings suggest that improvement in the clinical outcome did not correlate with improvement in tendon healing, and this can be partially explained by the characteristics of the patients included in the study. Different factors such as the age of the patient, large tear size, initial tendon retraction, initial muscle atrophy and the severity of preoperative fatty degeneration in the rotator cuff have been shown to determine the clinical outcome and integrity of the rotator cuff repair [4, 13].

Nevertheless, undesirable outcomes in some patients after the intervention limited our study, forcing its premature termination, and making it difficult to make conclusions with certainty, about the effectiveness of MSC treatment. Complications occurred in both study groups, suggesting that adverse effects are not caused by autologous MSCs but by the scaffold. OrthADAPT™ has characteristics close to those of an ideal scaffold for tendon engineering (the same native extracellular matrix and capability of cell seeding); however, its xenogenic nature, even after purification, decellularization and cross-linking of matrices, could not hide antigenic components and thus its immunologically inert nature could not be granted [25]. Although we can only speculate about the causes of these complications, the biological mechanisms of body response may be greatly influenced by the nature and/or composition of the extracellular matrix. In our study, the adverse effects were likely caused by induction of foreign-body reactions, as previously reported by other investigators in a canine model [35]. Although OrthADAPT™ has been used with favorable results in the reconstruction of ligaments, 191 adverse events reports were received by the Manufacturer and User Facility Device Experience (MAUDE) database (between 2009 and 2011) (Additional file 1). The manufacturer of OrthAdapt (Pegasus Biologics) was acquired by Synovis Life Technologies and the brand disappeared years ago from the market. However, Pegasus technicians founded Harbor MedTech, which continued to apply the same manufacturing technology to equine pericardial membranes marketed under the name of ARCHITECT (http://www.harbormedtech.com/architect/). This membrane is the same as OrthAdapt but its indications are focused on the treatment of skin problems; we have not identified adverse events on the MAUDE database. ARCHITECT is still available on the US market, but the company has not renewed the CE mark since more than a year ago (the CE is a certification mark that indicates conformity with health, safety and environmental protection standards for products sold within the European Economic Area).

In the current study, 61.5% of the implants failed, which shows that technically the use of xenogenic grafts is far more difficult than other treatment options and shows the need to improve techniques, to provide reinforcement of the repair site and adequate host regenerative responses.

It is also necessary to consider that, in general, the reporting of adverse effects in the literature is biased to favor positive results [18]. So, the benefits of the OrthADAPT™ implant as demonstrated in other applications might not be appropriate to its application in rotator cuff tears. In this sense, the anatomical characteristics of the rotator cuff, enclosed in a synovial cavity, could in some way facilitate the development of the observed localized immune reactions. The greater or lesser suitability of a scaffold to a particular treatment has been reflected in other studies showing the success of small intestine submucosa (SIS) augmentation in the repair of the Achilles and infraspinatus tendons, and its failure to repair massive chronic rotator cuff tears [20]. Recently, a prospective study demonstrated the benefits, in terms of better functional outcomes and structural reinforcement, of other xenograft membranes [24]. which highlights the relevance of bioscaffold choice in different musculoskeletal applications.

It is remarkable that most recent innovations in implant technology have made the systems more user-friendly but that no significant advantages of the implant survival are seen in very long periods of follow up. The lengths, diameter, region of implantation and merged or submerged healing were not significant predictors, but both xenogenic and allogenic grafts showed lower survival rates than autogenic graft,s with a HR of 4.74 [39].

As allogeneic or xenogenic tissue might not be an option for every indication, in order to achieve a less exaggerated immune reaction, preclinical experiments could add valuable information on the refinement of decellularization or processing methods for xenogeneic tissue. Interestingly, both processed human and bovine pericardial patches lead to greater immune cell proliferation [40]. It has been suggested that even decellularized collagen matrix retains some remnant immunogenicity, perhaps in the form of residual highly immunogenic dendritic cells that may be more resistant to the decellularization process. The resultant inflammatory process could lead to foreign-body reaction and massive fibrosis [41] as demonstrated in our case.

## Conclusion

In summary, our study showed preliminary inconclusive clinical outcomes in the patients treated with MSCs. Adverse events revealed the need for further approaches using scaffolds of a different nature or perhaps no scaffolds, in the context of small joints. Moreover, there is a need for further controlled studies and additional research on site-specific scaffold behavior, as animal models do not provide all the information needed when undertaking human research.

## Additional file

Additional file 1: Report of Orthadapt adverse events. (XLS 269 kb)

## Data Availability

Not applicable.

## Abbreviations

BM: Bone marrow
BMMCs: Bone marrow mononuclear cells
ECM: Extracellular matrix
GMP: Good Manufacturing Practice
MRI: Resonance imaging
MSCs: Mesenchymal stem cells
NSAID’s: Nonsteroidal anti-inflammatory drugs
VAS: Visual analogue pain scale

## References

[1] Anderson K, Boothby M, Aschenbrener D, van Holsbeeck M (2006). Outcome and structural integrity after arthroscopic rotator cuff repair using 2 rows of fixation: minimum 2-year follow-up. Am J Sports Med.

[2] Barber FA, Herbert MA, Boothby MH (2008). Ultimate tensile failure loads of a human dermal allograft rotator cuff augmentation. Arthroscopy.

[3] Beitzel K, Solovyova O, Cote MP, Apostolakos J, Russell RP, McCarthy MB (2013). The future role of mesenchymal stem cells in the management of shoulder disorders. Arthroscopy.

[4] Bjornsson HC, Norlin R, Johansson K, Adolfsson LE (2011). The influence of age, delay of repair, and tendon involvement in acute rotator cuff tears: structural and clinical outcomes after repair of 42 shoulders. Acta Orthop.

[5] Butler DL, Juncosa-Melvin N, Boivin GP, Galloway MT, Shearn JT, Gooch C (2008). Functional tissue engineering for tendon repair: a multidisciplinary strategy using mesenchymal stem cells, bioscaffolds, and mechanical stimulation. J Orthop Res.

[6] Caplan AI (2005). Review: mesenchymal stem cells: cell-based reconstructive therapy in orthopedics. Tissue Eng.

[7] Carbonel I, Martinez AA, Aldea E, Ripalda J, Herrera A (2013). Outcome and structural integrity of rotator cuff after arthroscopic treatment of large and massive tears with double row technique: a 2-year followup. Adv Orthop.

[8] Clarke AW, Alyas F, Morris T, Robertson CJ, Bell J, Connell DA (2011). Skin-derived tenocyte-like cells for the treatment of patellar tendinopathy. Am J Sports Med.

[9] Clement ND, Nie YX, McBirnie JM (2012). Management of degenerative rotator cuff tears: a review and treatment strategy. Sports Med Arthrosc Rehabil Ther Technol.

[10] Constant CR, Murley AH. A clinical method of functional assessment of the shoulder. Clin Orthop Relat Res. 1987;214:160–4. 3791738

[11] Chen PM, Yen ML, Liu KJ, Sytwu HK, Yen BL (2011). Immunomodulatory properties of human adult and fetal multipotent mesenchymal stem cells. J Biomed Sci.

[12] Cho CH, Lee SM, Lee YK, Shin HK (2014). Mini-open suture bridge repair with porcine dermal patch augmentation for massive rotator cuff tear: surgical technique and preliminary results. Clin Orthop Surg.

[13] Deniz G, Kose O, Tugay A, Guler F, Turan A (2014). Fatty degeneration and atrophy of the rotator cuff muscles after arthroscopic repair: does it improve, halt or deteriorate?. Arch Orthop Trauma Surg.

[14] Derwin KA, Baker AR, Spragg RK, Leigh DR, Iannotti JP (2006). Commercial extracellular matrix scaffolds for rotator cuff tendon repair. Biomechanical, biochemical, and cellular properties. J Bone Joint Surg Am.

[15] Ejnisman B, Andreoli CV, Soares BG, Fallopa F, Peccin MS, Abdalla RJ, et al. Interventions for tears of the rotator cuff in adults. Cochrane Database Syst Rev. 2004:CD002758. 10.1002/14651858.CD002758.pub2. 10.1002/14651858.CD002758.pub2 14973989

[16] Ellera Gomes João L., da Silva Ricardo Canquerini, Silla Lúcia M. R., Abreu Marcelo R., Pellanda Roberto (2011). Conventional rotator cuff repair complemented by the aid of mononuclear autologous stem cells. Knee Surgery, Sports Traumatology, Arthroscopy.

[17] Galatz LM, Ball CM, Teefey SA, Middleton WD, Yamaguchi K (2004). The outcome and repair integrity of completely arthroscopically repaired large and massive rotator cuff tears. J Bone Joint Surg Am.

[18] Golder S, Loke YK, Wright K, Norman G (2016). Reporting of adverse events in published and unpublished studies of health care interventions: a systematic review. PLoS Med.

[19] Havlas V, Kotaska J, Konicek P, Trc T, Konradova S, Koci Z (2015). Use of cultured human autologous bone marrow stem cells in repair of a rotator cuff tear: preliminary results of a safety study. Acta Chir Orthop Traumatol Cechoslov.

[20] Iannotti JP, Codsi MJ, Kwon YW, Derwin K, Ciccone J, Brems JJ (2006). Porcine small intestine submucosa augmentation of surgical repair of chronic two-tendon rotator cuff tears. A randomized, controlled trial. J Bone Joint Surg Am.

[21] Itoi E (2013). Rotator cuff tear: physical examination and conservative treatment. J Orthop Sci.

[22] Lafosse L, Brozska R, Toussaint B, Gobezie R (2007). The outcome and structural integrity of arthroscopic rotator cuff repair with use of the double-row suture anchor technique. J Bone Joint Surg Am.

[23] Lafosse L, Brzoska R, Toussaint B, Gobezie R (2008). The outcome and structural integrity of arthroscopic rotator cuff repair with use of the double-row suture anchor technique. Surgical technique. J Bone Joint Surg Am.

[24] Lederman ES, Toth AP, Nicholson GP, Nowinski RJ, Bal GK, Williams GR (2016). A prospective, multicenter study to evaluate clinical and radiographic outcomes in primary rotator cuff repair reinforced with a xenograft dermal matrix. J Shoulder Elb Surg.

[25] Lovati AB, Bottagisio M, Moretti M (2016). Decellularized and engineered tendons as biological substitutes: a critical review. Stem Cells Int.

[26] Mirza A, Hyvelin JM, Rochefort GY, Lermusiaux P, Antier D, Awede B (2008). Undifferentiated mesenchymal stem cells seeded on a vascular prosthesis contribute to the restoration of a physiologic vascular wall. J Vasc Surg.

[27] Patte D. Classification of rotator cuff lesions. Clin Orthop Relat Res. 1990;250:81–6. 2323151

[28] Petrie Aronin CE, Tuan RS (2010). Therapeutic potential of the immunomodulatory activities of adult mesenchymal stem cells. Birth Defects Res C Embryo Today.

[29] Ruiz-Moneo P, Molano-Munoz J, Prieto E, Algorta J (2013). Plasma rich in growth factors in arthroscopic rotator cuff repair: a randomized, double-blind, controlled clinical trial. Arthroscopy.

[30] Sheng Z, Fu X, Cai S, Lei Y, Sun T, Bai X (2009). Regeneration of functional sweat gland-like structures by transplanted differentiated bone marrow mesenchymal stem cells. Wound Repair Regen.

[31] Smith RK, Werling NJ, Dakin SG, Alam R, Goodship AE, Dudhia J (2013). Beneficial effects of autologous bone marrow-derived mesenchymal stem cells in naturally occurring tendinopathy. PLoS One.

[32] Squillaro Tiziana, Peluso Gianfranco, Galderisi Umberto (2016). Clinical Trials with Mesenchymal Stem Cells: An Update. Cell Transplantation.

[33] Thomazeau H, Rolland Y, Lucas C, Duval JM, Langlais F (1996). Atrophy of the supraspinatus belly. Assessment by MRI in 55 patients with rotator cuff pathology. Acta Orthop Scand.

[34] Tornero-Esteban P, Hoyas JA, Villafuertes E, Rodriguez-Bobada C, Lopez-Gordillo Y, Rojo FJ (2015). Efficacy of supraspinatus tendon repair using mesenchymal stem cells along with a collagen I scaffold. J Orthop Surg Res.

[35] Turner TM, Urban RM, Hall DJ, Dahlmeier EL (2009). Achilles tendon repair augmented with a decellularized porcine dermal graft compared to two other collagen xenografts. Arthroscopy Society For Biomaterials Annual Meeting and Exposition.

[36] Veeger HE, van der Helm FC (2007). Shoulder function: the perfect compromise between mobility and stability. J Biomech.

[37] Walch G, Marechal E, Maupas J, Liotard JP (1992). Surgical treatment of rotator cuff rupture. Prognostic factors. Rev Chir Orthop Reparatrice Appar Mot.

[38] Yamaguchi K, Levine WN, Marra G, Galatz LM, Klepps S, Flatow EL (2003). Transitioning to arthroscopic rotator cuff repair: the pros and cons. Instr Course Lect.

[39] Zinser MJ, Randelzhofer P, Kuiper L, Zöller JE, De Lange GL (2013). The predictors of implant failure after maxillary sinus floor augmentation and reconstruction: a retrospective study of 1045 consecutive implants. Oral Surg Oral Med Oral Pathol Oral Radiol.

[40] Rieder E, Steinacher-Nigisch A, Weigel G (2016). Human immune-cell response towards diverse xenogeneic and allogeneic decellularized biomaterials. Int J Surg.

[41] Cichaa I, Rüfferb A, Cesnjevarb R, Glöcklerc M, Agaimyd A, Daniela WG, Garlichsa CD, Dittrich S (2011). Early obstruction of decellularized xenogenic valves in pediatric patients involvement of inflammatory and fibroproliferative processes. Cardiovasc Pathol.

